# Multiplex analysis of inflammatory proteins associated with risk of coronary artery disease in type‐1 diabetes patients

**DOI:** 10.1002/clc.24143

**Published:** 2023-10-11

**Authors:** Carol Beatty, Katherine P. Richardson, Paul M. H. Tran, Khaled B. Satter, Diane Hopkins, Melissa Gardiner, Ashok Sharma, Sharad Purohit

**Affiliations:** ^1^ School of Medicine Medical College of Georgia Augusta Georgia USA; ^2^ School of Medicine Center for Biotechnology and Genomic Medicine, Medical College of Georgia, Augusta University Augusta Georgia USA; ^3^ Department of Internal Medicine Yale School of Medicine New Haven Connecticut USA; ^4^ Department of Pathology National Institutes of Health Bethesda Maryland USA; ^5^ Department of Obstetrics and Gynecology Medical College of Georgia, Augusta University Augusta Georgia USA

**Keywords:** biomarkers, chronic inflammation, coronary artery disease, cytokines, type‐1 diabetes

## Abstract

**Background:**

Chronic uncontrolled hyperglycemia, a precursor to chronic low‐grade inflammation, is a leading cause of coronary artery disease (CAD) due to plaque buildup in type‐1 diabetes (T1D) patients. We evaluated levels of 22 inflammatory markers in cross‐sectional serum samples from 1222 subjects to evaluate their potential as risk factors for CAD in T1D patients.

**Hypothesis:**

Circulating levels of markers of inflammation may be the risk factors for incident CAD.

**Methods:**

The T1D subjects were divided into two groups: those without CAD (*n* = 1107) and with CAD (*n* = 115). Serum levels of proteins were assayed using multiplex immunoassays on a Luminex Platform. Differences between the two groups were made by univariate analysis. Multivariate logistic regression was used to ascertain the potential of proteins as risk factors for CAD. Influence of age, duration of diabetes, sex, hypertension, and dyslipidemia was determined in a stepwise manner. Serum levels of 22 proteins were combined into a composite score using Ridge regression for risk‐based stratification.

**Results:**

Mean levels of CRP, IGFBP1, IGFBP2, insulin‐like growth factors binding protein‐6 (IGFBP6), MMP1, SAA, sTNFRI, and sTNFRII were elevated in CAD patients (*n* = 115) compared to T1D patients without CAD (nCAD, *n* = 1107). After adjusting for age, duration of diabetes, sex, hypertension, and dyslipidemia, higher levels of sTNFRI (odds ratio [OR] = 2.18, 1.1 × 10^−3^), sTNFRII (OR = 1.52, 1 × 10^−2^), and IGFBP6 (OR = 3.62, 1.8 × 10^−3^) were significantly associated with CAD. The composite score based on Ridge regression, was able to stratify CAD patients into low, medium, and high‐risk groups.

**Conclusions:**

The results show activation of the TNF pathway in CAD patients. Evaluating these markers in serum can be a potential tool for identifying high‐risk T1D patients for intensive anti‐inflammatory therapeutic interventions.

## Graphical abstract

Serum levels of 22 proteins were measured in type‐1 diabetes (T1D) patients with and without coronary artery disease (CAD) in a high‐throughput manner using multiplex immunoassays. Machine learning‐based scores can stratify CAD patients into low, medium, and high‐risk groups. Our findings have translational potential to identify T1D patients at higher risk of CAD.

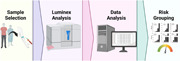

## 1 BACKGROUND

Coronary artery disease (CAD) is a complication and leading cause of mortality in type‐1 diabetes (T1D) patients.^1^ Resulting atherosclerosis, due to plaque buildup in the vessels, is linked with ischemic stroke and myocardial infarction.^1^ Studies are now showing an established link between hyperglycemia, CAD, and diagnostic HbA_1c_ as predictors for cardiovascular disease (CVD) in T1D patients.^2^, ^3^ World Health Organization reports show increased blood glucose levels account for 20% of cardiovascular‐related deaths globally.^4^ Increased dysglycemia resulted in 11%–16% increase in rates of CVD events for every 1% increase in HbA1c levels.^2^, ^3^

The Diabetes Control and Complications Trial (DCCT) conducted a longitudinal clinical trial administering an intensive therapy that consisted of multiple daily insulin injections or an external insulin pump to maintain normoglycemic levels.^5^ After following participants for 17 years, they found a mean of 6.5 years of routine intensive therapy reduced the incidence of CVD by 30%–42%.^5^ This intensive intervention was suggested to induce beneficial long‐term vascular changes in the early phase of disease that would have long‐lasting effects.^5^, ^6^ Another study in 2019 focused on measuring the prevalence of cardiac autoantibodies (AAbs) in the DCCT.^7^ They found patients with HbA1c of ≥9.0% had higher cardiac AAb levels.^7^ Patients with ≥2 AAbs showed increased hs‐CRP levels, which are linked to inflammation.^7^ The findings from these DCCT investigations propose HbA1c and cardiac AAbs as potential risk factors for the development of CVD. However, HbA1c and cardiac AAbs can also be associated with multiple complications of diabetes making it challenging to directly link their role in CAD progression. Several clinical markers are risk factors for CAD in T1D patients^8^; however, these markers lack in identifying and stratifying T1D patients into risk groups based on their inflammatory protein profiles.

In the hopes of improving risk stratification for T1D patients at increased risk of developing CAD, further research on serum inflammatory markers is required.^9^ One of the underlying features of CAD in diabetes is chronic low‐grade inflammation, which leads to atherogenic changes in arteries.^10^ Previous work has linked markers sTNFRI and sTNFRII^11^ to CAD in T1D patients; however, we aimed to explore several other markers linked to low‐grade inflammation to provide a targeted approach for future clinical application. It is for these reasons we investigated and report our results on serum levels of inflammatory markers and their potential as risk factors for CAD in a cross‐sectional cohort of T1D patients.

## 2 METHODS

### 2.1 Experimental design and study population

Serum samples (*n* = 1107) for this cross‐sectional study were obtained from Caucasian subjects recruited into the Phenome and Genome of Diabetes Autoimmunity study between 2002 and 2010. These subjects attended the Augusta University (AU) Medical Center and/or endocrinology clinics in the Augusta or metro‐Atlanta areas of Georgia. Medical history, clinical, and demographic profiles for T1D subjects were obtained from medical chart review (Table 1). All study participants or their legal representatives gave written informed consent before enrolling in the study. The study was carried out according to The Code of Ethics of the World Medical Association (Declaration of Helsinki, 1997) and was approved by the institutional review board at AU.

**Table 1:** Demographic and clinical information on the T1D patients with CAD and without nCAD.

Characteristics	nCAD (*n* = 1107)	CAD (*n* = 115)	*p* Value
Gender, *n* (%)			
Male	546	56	
Female	561	59	.9761*
Age (years)	20.6 + 13.2	59.65 + 12.9	5.5 × 10^−64^
Median age (range, years)	16.2 (2.6–73.7)	59.5 (14.6–87.3)	1.2 × 10^−60^ **
Median age of diagnosis (range, years)	10 (0–60)	26.8 (1–78)	3.8 × 10^‐28^ **
Duration of T1D (years)	8.6 + 8.7	31.9 + 14.4	4.5 × 10^−34^
UACR (ug/mg)	8.79 (1.23–5642.9)	11.79 (1.67–4782.8)	.000241**
BUN (mg/dL)	12.7 + 3.4	20.6 + 10.6	1.4 × 10^−9^
HbA1c (%, NGSP)	8.1 + 1.5	7.9 + 1.1	.0951
	7.9 (5.4–14.6)	7.6 (5.7–11.6)	.2**
HbA1c (mmol/mol, IFCC)	65.4 + 16.0	63.0 + 12.5	.0951
	63.6 (36.1–136.4)	59.2 (38.4–103.82)	.2**
Total cholesterol (mg/dL)	166.7 + 30.3	161.9 + 36.82	.246
LDL (mg/dL)	93.2 + 24.9	85.5 + 32.4	.03761
Triglycerides	95.2 + 69.4	108.5 + 79.1	.1415
HDL	54.8 + 15.8	54.5 + 16.4	.8752
Blood pressure (mmHg)			
Systolic BP	115.2 + 10.8	127.3 + 12.1	8.7 × 10^−15^
Diastolic BP	70.4 + 7.1	71.9 + 5.8	.0197
MAP	85.3 + 7.7	90.4 + 6.4	1.7 × 10^−10^
Other complications			
Nephropathy (*n*)	0	25	
Retinopathy (*n*)	0	57	
Hypertension (*n*)	0	69	
Dyslipidemia (*n*)	0	14	
CABG (*n*)	0	46	
MI (*n*)	0	34	
Angioplasty stent (*n*)	0	64	

Abbreviations: BUN, blood urea nitrogen; CAD, coronary artery disease; CABG, coronary artery bypass graft; HDL, high density lipoprotein; IFCC, international federation of clinical chemistry working group; LDL, low density lipoprotein; MAP, mean arterial pressure; MI, myocardial infarction; nCAD, no coronary artery disease; T1D, type‐1 diabetes; NGSP, national glycohemoglobin standardization program; UACR, urine albumin‐creatinine ratio.

* *χ* ^2^ test, all other *p* values are from *t*‐tests.

** Kruskall–Wallis test.

Venous blood was collected in clot activator tubes and allowed to clot at room temperature for 30 minutes before centrifugation at 3000*g*. Separated serum was then aliquoted into wells of a 96‐well plate to create a master plate. Individual daughter plates were then created by aliquoting 5–10 µL of serum from this master plate. All master and daughter plates were stored at −80°C until use.

### 2.2 Laboratory measurements

Fluorescent bead‐based immune‐assays for IL1Ra, IL8, MCP‐1, MIP‐1β, CRP, SAA, MMP1, MMP2, MMP9, sgp130, sICAM1, sVCAM1, sIL2Rα, sIL6R, sTNFRI, sTNFRII, sEGFR, IGFBP1, IGFBP2, IGFBP3, insulin‐like growth factors binding protein‐6 (IGFBP6) and tPAI1 (Millipore Inc.), were used to measure serum levels. Briefly, serum samples were incubated with antibody‐coated microspheres, followed by biotinylated detection antibody. Detection of the proteins was accomplished by incubation with phycoerythrin‐labeled streptavidin. The resultant bead immuno‐complexes were then read on a FLEXMAP3D (Luminex) with the instrument settings recommended by the manufacturer.

The captured median fluorescence intensity (MFI) data was subjected to our quality control steps. Briefly, wells with individual bead counts below 30 or bead count coefficient of variation (CV) above 200 were flagged for exclusion. Replicate wells with CV ≥ 25% were excluded from further analyses. The standard concentration and MFI were log2 transformed before regression. Protein concentrations were estimated using a regression fit to the standard curve with serial dilution of known concentration for each protein.^12^, ^13^

### 2.3 Construction of inflammation score

Serum levels of all 22 proteins were combined into a composite score by Ridge regression (R package “glmnet”).^14^ The algorithm combines the serum levels of multiple proteins in a linear combination by using the sum of the squares of the coefficients of individual proteins generated in the model based on a penalty term.^15^ We fit all 22 proteins to the CAD as outcome using a least absolute shrinkage and selection operator by setting alpha = 0 in the cv. glmnet function to generate a linear predictor (Lp) score (Supporting Information: Figure 2A). We manually removed the data for six proteins that were contributing the lowest weight to the Lp (Supporting Information: Figure 2B).

After the selection of proteins for the final model, we ran ridge regression using cv. glmnet function to obtain a Lp for 22‐proteins (Lp22) and 18‐proteins (Lp18). The composite score was then used to calculate the odds ratios (OR) of having CAD for the upper four quintiles using the first quintile as a reference.

### 2.4 Statistical analysis

For nominal data, count and percentage are reported, whereas for normally distributed variables, the mean and standard deviation are reported. Differences were tested utilizing the *χ* ^2^ test or *t*‐test. Median and range are presented for nonnormal variables. Differences were tested using the Kruskall–Wallis test. Potential univariate differences between T1D patients with (CAD) and without CAD (nCAD), were examined using boxplots and *t*‐test. Pairwise correlations between individual proteins were determined using the Pearson correlation coefficient and presented as a heatmap with hierarchical clustering. The association between the serum levels of each candidate molecule and age and T1D duration was determined using linear regression including sex and disease status as covariates.

Before any statistical analysis, protein concentration data was log2 transformed to follow the normal distribution. For the generation of the figures, the data was then back‐transformed to natural units. For all association analysis, log2 transformed concentration data were scaled to unit standard deviation. The potential relationship between CAD and inflammatory proteins was evaluated using logistic regression models where the presence/absence of CAD was incorporated as a dependent variable. Covariates: age, duration of diabetes, sex, hypertension (HTN), and dyslipidemia were adjusted in separate models in the logistic regression.

We removed T1D subjects with diabetic foot ulcers as well as complications such as nephropathy, neuropathy, and retinopathy (*n* = 328). These complications of diabetes are commonly linked to chronic inflammation and could interfere as distractors. The average last three HbA1c measurements and % HbA1c were converted into IFCC units as required by the American Diabetes Association for harmonized reporting of HbA1c values. Information about past history of CAD, myocardial infarction or coronary artery bypass graft, or angioplasty for placement of stents was utilized to group T1D patients with these conditions into CAD group.

Risk of CAD with increasing serum levels was evaluated by dividing serum level for each protein into five quintiles containing 20% CAD patients in each quintile (20th percentile). The cutoff protein levels from CAD patients were then used to count nCAD and CAD subjects in each quintile. The first quintile was used as reference and OR for CAD was calculated for the upper four quintiles. Pearson's *χ* ^2^ test with Yates' continuity correction was used to calculate the ORs. The *χ* ^2^ test for trend in proportions was used to calculate the *p* value of overall trend.

All *p* values were two‐tailed and a *p* < .05 was considered statistically significant. All statistical analyses were performed using the R language and environment for statistical computing (v 4.0.3; R Foundation for Statistical Computing; www.r-project.org).

## 3 RESULTS

### 3.1 Description of the study population

In this cross‐sectional study, serum samples from patients with T1D (*n* = 1107) were analyzed (Table 1). The samples were divided into two groups: patients with CAD (CAD, *n* = 115) and those without CAD (nCAD, *n* = 1107). On average, the patients diagnosed with CAD were older (59.7 vs. 20.6 years). Despite being diagnosed later in life (median age of diagnosis 26.8 vs. 10.0 years), the CAD group had a longer duration of diabetes (31.9 vs. 8.6 years). Patients with CAD had higher levels of urine albumin‐creatinine ratio, blood urea nitrogen, systolic blood pressure, and mean arterial pressure, but had lower total cholesterol and LDL levels. HTN and dyslipidemia were present in both groups (Table 1).

### 3.2 Relationships of inflammatory proteins in CAD patients

**Figure 1:**
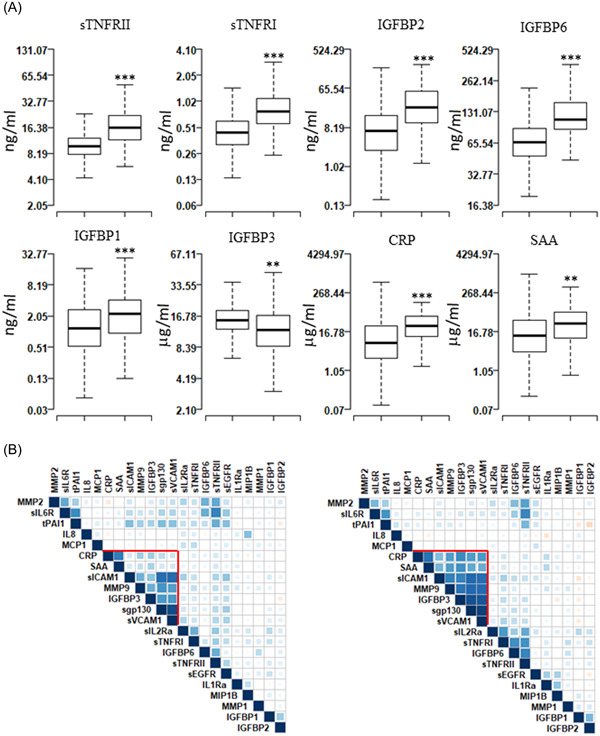
Serum levels of proteins in type‐1 diabetes patient (T1D) with no coronary artery disease (nCAD) and those with coronary artery disease (CAD) and pairwise correlations among proteins. (A) Boxplots showing the measured levels of selected proteins in nCAD group (*n* = 1107) and CAD (*n* = 115) group. Boxplots for all proteins are presented in Supporting Information: Figure 1, mean ± SD values are presented in supplementary Table 1. (B) Heatmaps showing the pairwise correlation coefficients in nCAD (left) and CAD (right) patients. The correlation coefficients are clustered based on supervised hierarchical clustering. The color density represents the strength of correlation (*r*) values and the size of the squares represent the significance. *** *p* < .000001, ** *p* < .01, *p < .05.

Serum levels of 22 proteins (IL1Ra, IL8, MCP‐1, MIP‐1β, CRP, SAA, MMP1, MMP2, MMP9, sgp130, sICAM1, sVCAM1, sIL2Rα, sIL6R, sTNFRI, sTNFRII, sEGFR, IGFBP1, IGFBP2, IGFBP3, IGFBP6, and tPAI1) were measured in all samples, *n* = 1222 (Figure 1A). The mean expression levels between the groups showed highly significant differences for five proteins (sTNFRI, sTNFRII, IGFBP2, IGFBP6, IGFBP1; *p* < 1 × 10^−4^) and moderately significant differences for three proteins (IGFBP3, CRP, SAA; *p* < .05) (Supporting Information: Table 1 and Supporting Information: Figure 1). Correlations between each pair of the 22 proteins were calculated separately for T1D patients without CAD (nCAD) and those with CAD (Figure 1B). A cluster of correlated proteins was defined by hierarchical clustering of the correlation matrix. The identified cluster included CRP, SAA, sICAM1, MMP9, IGFBP3, sgp130, and sVCAM1 (*r* = .97 [0.93–1.00]).

### 3.3 Influence of age, duration of diabetes, and sex on serum protein levels

Within the nCAD group, serum levels of proteins showed significant correlations with age, and duration of T1D (Supporting Information: Tables 2–3). Within nCAD group, significant differences existed between males and females, only CRP levels were significantly elevated in CAD group (Supporting Information: Table 4). These covariates were then adjusted for in the logistic regression analyses to rule out confounding factors in serum protein differences between CAD and nCAD patients (Table 2). Logistic regression analyses were carried out without any covariate adjustment (Model 1), with covariate adjustment for age (Model 2), with covariate adjustment for age and duration of diabetes (Model 3), and with adjustment for age, duration of diabetes, and sex (Model 4). Risk factors for CAD were considered in an additional logistic regression analysis that adjusted for HTN and dyslipidemia in addition to age, duration of diabetes, and sex (Model 5). In this analysis, six of the 22 serum proteins (CRP, IGFBP6, MMP2, sIL6R, sTNFRI, sTNFRII) showed significant differences between T1D patients with and without CAD both before and after adjusting for covariates (Table 2).

**Table 2:** Coronary artery disease is independently associated with per SD increase in serum levels, after adjusting for age at sample, duration of T1D, sex, glycemic control, hypertension, and dyslipidemia.

	Model 1	Model 2	Model 3	Model 4	Model 5
Proteins	OR (95% CI)	OR (95% CI)	OR (95% CI)	OR (95% CI)	OR (95% CI)
IL1Ra	1.09 (0.88–1.41)	1.03 (0.74–1.5)	0.97 (0.69–1.4)	0.97 (0.69–1.42)	0.75 (0.51–1.18)
IL8	0.95 (0.76–1.19)	1.34 (0.93–1.95)	1.43 (0.97–2.11)	1.43 (0.98–2.11)	1.34 (0.77–2.37)
MCP1	1.25 (1.01–1.56)*	1.29 (0.9–1.88)	1.18 (0.82–1.73)	1.18 (0.82–1.74)	1.03 (0.62–1.84)
MIP1B	0.85 (0.7–1.05)	1.07 (0.75–1.53)	1.01 (0.7–1.46)	1 (0.7–1.45)	0.73 (0.45–1.21)
CRP	1.98 (1.55–2.55)**	1.22 (0.87–1.77)	1.29 (0.92–1.87)	1.32 (0.93–1.92)	2.86 (1.38–6.51)***
IGFBP1	1.59 (1.29–1.97)****	1.21 (0.87–1.7)	1.09 (0.77–1.55)	1.1 (0.77–1.58)	1.01 (0.54–1.9)
IGFBP2	4.7 (3.4–6.63)**	1.11 (0.73–1.73)	0.99 (0.63–1.55)	0.99 (0.63–1.55)	0.93 (0.49–1.83)
IGFBP3	0.74 (0.64–0.86)****	0.78 (0.61–1.02)	0.83 (0.64–1.11)	0.83 (0.64–1.11)	1.88 (0.76–5.05)
IGFBP6	5.08 (3.75–7.03)**	2.37 (1.56–3.67)****	2.16 (1.41–3.38)****	2.31 (1.47–3.74)****	3.62 (1.67–8.5)***
MMP1	1.7 (1.38–2.11)**	1.25 (0.88–1.77)	1.23 (0.86–1.77)	1.24 (0.86–1.78)	1.38 (0.83–2.31)
MMP2	1.57 (1.21–2.07)***	1.53 (1.06–2.3)	1.27 (0.88–1.91)	1.27 (0.88–1.91)	2.82 (1.36–6.26)***
MMP9	0.78 (0.66–0.94)***	0.82 (0.61–1.11)	0.82 (0.61–1.12)	0.82 (0.61–1.12)	0.95 (0.54–1.82)
SAA	1.56 (1.25–1.97)	1.05 (0.73–1.5)	1.12 (0.78–1.61)	1.13 (0.78–1.64)	1.76 (0.98–3.22)
sEGFR	0.81 (0.69–0.97)*	1.11 (0.82–1.54)	1.03 (0.76–1.43)	1.03 (0.75–1.43)	0.95 (0.63–1.53)
sgp130	1.14 (0.91–1.5)	0.91 (0.67–1.25)	0.91 (0.67–1.27)	0.91 (0.67–1.27)	1.91 (0.9–4.14)
sICAM1	1.28 (1.03–1.61)*	1.27 (0.92–1.78)	1.29 (0.93–1.8)	1.29 (0.93–1.81)	1.57 (0.89–2.83)
sIL2Ra	1.28 (1.05–1.56)*	1.5 (1.13–1.99)***	1.46 (1.09–1.96)*	1.46 (1.09–1.96)*	1.47 (0.96–2.27)
sIL6R	1.03 (0.86–1.3)	1.15 (0.8–1.72)	1.12 (0.77–1.71)	1.13 (0.77–1.71)	2.27 (1.06–5.07)*
sTNFRI	3.3 (2.6–4.25)**	2.36 (1.75–3.26)	2.3 (1.71–3.17)	2.33 (1.73–3.23)	2.18 (1.4–3.59)***
sTNFRII	4.17 (3.08–5.74)**	1.54 (1.19–2.13)***	1.46 (1.13–2)***	1.46 (1.13–2)***	1.52 (1.1–2.25)*
sVCAM1	1.06 (0.86–1.35)	0.98 (0.72–1.36)	1.04 (0.75–1.43)	1.04 (0.75–1.43)	1.26 (0.61–2.65)
tPAI1	0.81 (0.69–0.95)***	1.15 (0.85–1.61)	1.17 (0.85–1.65)	1.17 (0.85–1.66)	1.6 (0.91–2.83)

*Note*: Values presented are OR, 95% CI. Model 1: protein concentration only, Model 2: protein concentration + Age, Model 3: Model 2 + Dx_Age, Model 4: Model 3 + Sex, Model 5: Model 4 + Hypertension + Dyslipidemia.

Abbreviations: CI, confidence interval; OR, odds ratio; T1D, type‐1 diabetes.

* *p* < .05

** *p* < 1 × 10^‐4^

*** *p* < .01

**** *p* < .001.

### 3.4 CAD is associated with increase in serum levels of inflammatory proteins

**Figure 2:**
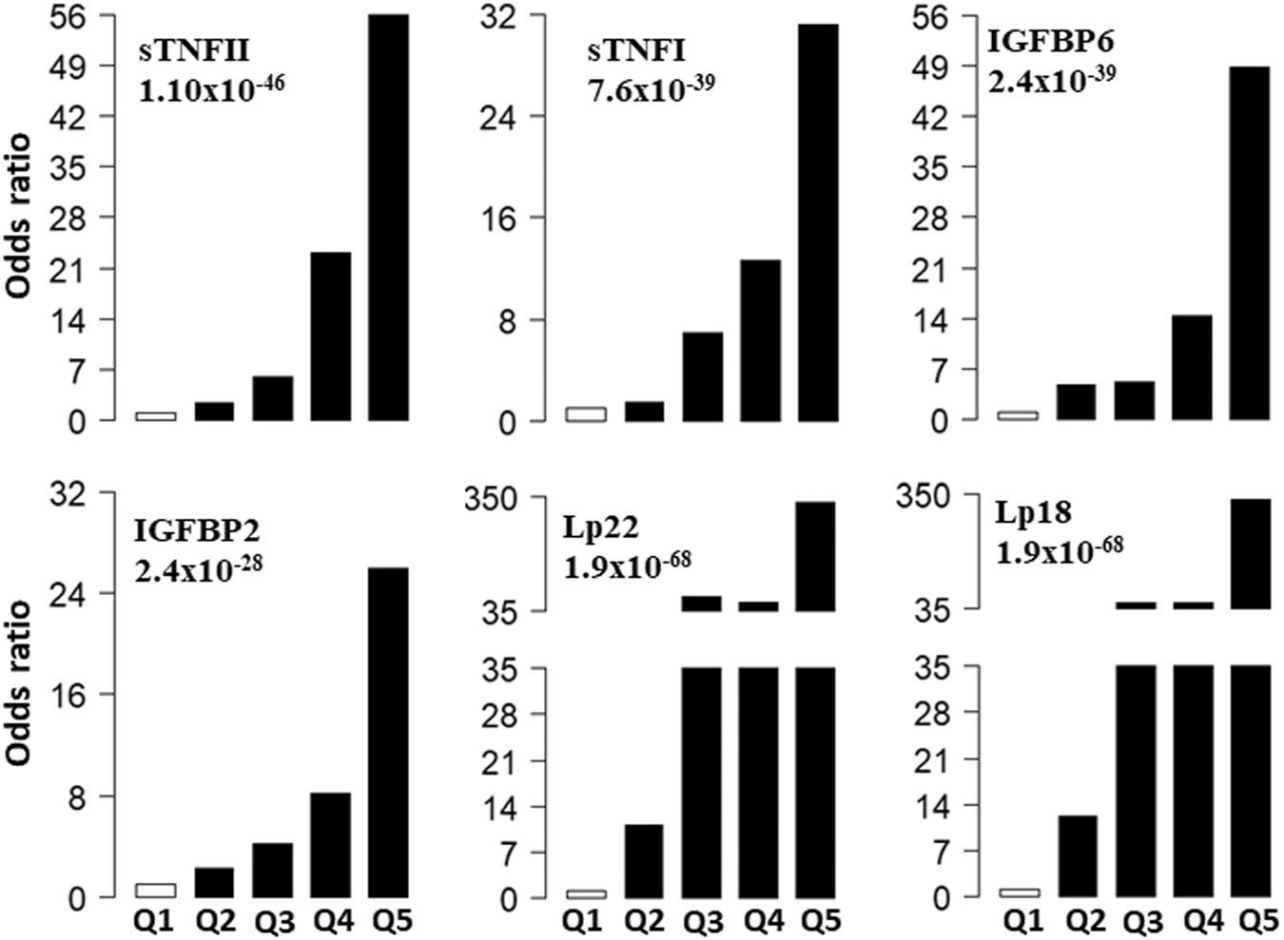
Association of serum level of proteins after dividing them into 20th percentile groups (quintiles). Odds ratios (OR) associated with each of the top four quintiles compared to the bottom first quintile for each of the 12 individual proteins. The open bar represents the first quintile was used as reference (OR = 1). From left to right, each of the other four solid bars represents the second to fifth quintile (20% of the coronary artery disease patients). ORs associated with the risk scores calculated based on different combinations of 22 (Lp22) and 18 (Lp18) proteins using ridge regression. Values presented on vertical axes (*y*‐axis) are ORs (additional details are presented in Table 3). Quintile‐based groups are presented on *x*‐axis.

To determine the relationship between the incidence of CAD and the serum levels of proteins, we calculated ORs for protein concentrations divided into quintiles (Table 3 and Figure 2). The serum concentration of each protein in CAD patients was divided into five quintiles of 20% subjects and cutoff concentration values. Using these cutoff values, protein levels from T1D patients without CAD (nCAD) were then distributed into five groups based on the quintiles (Q1‐5). The first quintile was used as a reference to calculate ORs for the second, third, fourth, and fifth quintiles. The ORs with 95% CI and *p* values are presented for each serum protein in Table 3. CAD was associated with 16 out of the 22 serum proteins. The strongest association was seen with sTNFRII (1.1 × 10^−46^), which has ORs of 55.93, 23.03, 5.96, and 2.25 for the fifth, fourth, third, and second quintiles. The second strongest association was seen with sTNFRI (7.60 × 10^−39^), which has ORs of 31.27, 12.58, 6.88, and 1.41 for the fifth to second quintiles, respectively. The next strongest associations were seen in IGFBP6 and IGFBP2 (Figure 2 and Supporting Information: Figure 2).

**Table 3:** OR (95% CI) of having coronary artery disease based on serum levels divided into quintile (20th percentile) categories.

	Quintile 2	Quintile 3	Quintile 4	Quintile 5	
Protein	OR (95% CI)	OR (95% CI)	OR (95% CI)	OR (95% CI)	*p* Value^a^
IL1Ra	0.73 (0.36–1.47)	0.98 (0.49–1.98)	1.07 (0.53–2.17)	1.13 (0.56–2.29)	.30
IL8	1.45 (0.73–2.89)	1.17 (0.59–2.32)	1.3 (0.65–2.58)	1.04 (0.53–2.06)	.85
MCP1	1.54 (0.79–2.99)	1.52 (0.78–2.95)	1.69 (0.87–3.28)	1.68 (0.86–3.26)	.09
MIP1B	1.33 (0.67–2.63)	1.38 (0.7–2.73)	0.87 (0.44–1.7)	1.02 (0.52–2)	.48
CRP	3.45 (1.82–6.52)	4.23 (2.23–8.04)	6.44 (3.34–12.41)	3.94 (2.08–7.47)	9.6 × 10^−9^
IGFBP1	1.46 (0.8–2.66)	1.79 (0.98–3.27)	2.86 (1.55–5.27)	2.44 (1.33–4.48)	.0003
IGFBP2	2.24 (1.23–4.07)	4.22 (2.3–7.74)	8.18 (4.37–15.33)	25.98 (12.71–53.11)	2.4 × 10^−^^28^
IGFBP3	0.31 (0.15–0.62)	0.16 (0.08–0.32)	0.11 (0.06–0.22)	0.15 (0.08–0.30)	1.30 × 10^−^^8^
IGFBP6	4.65 (2.54–8.51)	5.19 (2.83–9.52)	14.32 (7.51–27.31)	48.9 (22.47–106.39)	2.4 × 10^−^^39^
MMP1	1.6 (0.88–2.91)	2.65 (1.44–4.86)	2.34 (1.28–4.28)	5.38 (2.87–10.08)	1.2 × 10^−7^
MMP2	2.03 (1.12–3.69)	1.19 (0.66–2.15)	2.5 (1.37–4.57)	2.26 (1.24–4.12)	.0045
MMP9	0.87 (0.46–1.65)	1.06 (0.55–2.02)	0.75 (0.4–1.42)	0.58 (0.31–1.09)	.079
SAA	2.06 (1.1–3.87)	2.24 (1.19–4.21)	3.28 (1.73–6.22)	3.05 (1.61–5.77)	4.9 × 10^−5^
sEGFR	1.24 (0.66–2.31)	0.77 (0.42–1.42)	0.44 (0.24–0.8)	0.49 (0.27–0.9)	.0003
sgp130	2.41 (1.27–4.57)	1.69 (0.9–3.18)	2.2 (1.16–4.16)	2.78 (1.46–5.28)	.0021
sICAM1	1.23 (0.65–2.32)	1.53 (0.81–2.89)	1.16 (0.62–2.18)	2.81 (1.46–5.39)	.0160
sIL2Ra	1.39 (0.76–2.54)	1.81 (0.99–3.32)	1.5 (0.82–2.74)	2.07 (1.13–3.81)	.0200
sIL6R	1.27 (0.7–2.32)	1.36 (0.74–2.49)	1.11 (0.61–2.02)	1.02 (0.56–1.86)	.92
sTNFRI	1.41 (0.78–2.55)	6.88 (3.69–12.84)	12.58 (6.53–24.22)	31.27 (14.79–66.11)	7.6 × 10^−39^
sTNFRII	2.25 (1.25–4.06)	5.96 (3.23–10.98)	23.03 (11.47–46.23)	55.93 (24.22–129.18)	1.1 × 10^−46^
sVCAM1	1.03 (0.55–1.94)	1.97 (1.03–3.77)	1.48 (0.78–2.82)	1.8 (0.94–3.44)	.0200
tPAI1	0.45 (0.23–0.87)	0.28 (0.15–0.54)	0.26 (0.14–0.5)	0.4 (0.21–0.77)	.005
Lp22	11.04 (4.81–25.33)	77.3 (25.9–230.75)	59.11 (21.14–165.26)	334.95 (56.52–1984.87)	1.9 × 10^−68^
Lp18	12.13 (5.26–27.96)	52.97 (19.4–144.66)	52.97 (19.4–144.66)	335.48 (56.61–1988)	1.9 × 10^−68^

*Note*: Quintile 1 was used as a reference.

Abbreviations: CI, confidence interval; Lp, linear predictor; OR, odds ratio.

^a^ Adjusted *p* value for Trend. Lp derived from a combination of 22 (Lp22) and 18 (Lp18) proteins.

### 3.5 Protein combinations stratify risk of CAD patients

Using ridge regression, we found that a combination of protein markers was strongly associated with having CAD. Lp values were derived from a combination of serum proteins and used to stratify risk for CAD. In the first Lp model (Lp), we combined serum levels of all 22 protein markers (Lp22), while in the second model, data from 18 proteins was combined together (Lp18). Both combined models had stronger associations with CAD than any individual serum protein. In Lp22 model, risk of CAD was highest in the fifth quintile (OR = 334.95), with the lowest risk was observed in the second quintile (OR = 11.04), the risk of CAD remained similar in the third and fourth quintiles (Table 3 and Figure 2). When data for proteins (IL1‐ra, IL‐8, MCP1, SAA, and sVCAM‐1) with minimal contributions were removed (Supporting Information: Figure 3), the OR's in all the quintiles remained similar (Figure 2). The Lp18 model had ORs of 335.48, 52.97, 52.97, and 12.13 for the fifth, fourth, third, and second quintiles (Figure 2 and Table 3). The composite Lps suggest different risk groups for CAD patients. Low‐risk CAD disease patients have serum levels in the first quintile (OR = 1), moderate risk in the second through fourth quintiles (OR < 59), and high risk in the fifth quintile (OR > 59).

## 4 DISCUSSION

We report that levels of eight serum proteins showed significant differences in T1D patients with and without CAD even when adjusting for covariates such as HTN, dyslipidemia, age, duration of diabetes, and sex (Figure 1A). These proteins have all been reported in the inflammation pathways associated with progression of T1D and its complications.^12^, ^15^, ^16^, ^17^, ^18^ These identified serum proteins could unlock improved methods to clinically treat and monitor T1D patients at risk of CAD. The correlated serum proteins or molecules in downstream signaling pathways could be targeted to develop novel pharmacotherapies utilized as agents for prophylaxis or clinical treatment. The identified cluster in Figure 1B (CRP, SAA, sICAM1, MMP9, IGFBP3, sgp130, and sVCAM1 (*r* = .97 [.93–1.00]) indicates that correlated changes in these immunologically active proteins could contribute synergistically to the pathogenesis of CAD due to a common upstream regulator.

Multivariable analysis accounting for multiple serum proteins is more significantly correlated to rates of CAD than analysis of individual serum markers. An Lp model was utilized to track the correlation of combinations of serum protein markers. This combined Lp model was able to accurately stratify patients by risk for CAD. Based on a patients' ORs for having CAD, the Lp model, Lp22, groups T1D patients into low risk (OR = 1), moderate risk (1 < OR < 59), or high risk (OR > 59) compared to T1D patients without CAD (Table 3). Since the model only requires serum proteins, patients' risk for CAD could be easily tracked with routine blood draws which are accessible and minimally invasive.

The correlations among serum protein levels and CAD support the role of inflammation in CAD pathogenesis. The proteins most significantly correlated with CAD after adjustment for covariates were sTNFRI, IGFBP6, and sTNFRII with *p* < .05 (Table 2). Both sTNFRI and sTNFRII are soluble cell surface receptors for TNF‐α, a proinflammatory cytokine.^19^ TNF‐α is responsible for the activation of macrophages, Th1 lymphocytes, and endothelial cells in atherosclerosis.^20^ Activated endothelial cells produce receptors and adhesion molecules for the abnormal migration of leukocytes into vessel walls, which in turn continue to release inflammatory cytokines.^21^ Similarly, IGFBP6 is associated with inflammation. Although the complete role of IGFBP6 is still largely unknown, it has recently been connected to cardiac remodeling and considered a biomarker for atherosclerotic plaques.^22^, ^23^

Our assay included markers of systemic inflammation and vascular function. The proteins involved in systemic inflammation, SAA and CRP, showed moderately increased levels in CAD versus nCAD patients. CRP has been widely utilized as an indicator of cardiovascular health.^24^, ^25^ While most proteins involved in vascular function did not show increased levels in T1D patients with CAD (sIL6R, sgp130, and sVCAM1), sICAM1 was significantly elevated after accounting for covariates (Table 2). Soluble intercellular adhesion molecule‐1 (sICAM‐1) is a soluble cell surface receptor that facilitates leukocyte adhesion and migration across the endothelium. Along with sVCAM1, it is known to be elevated in CAD in other studies as well.^26^ Both sICAM‐1 and sVCAM‐1 are upregulated via action of cytokines IL‐6 and TNF‐α. Interleukin‐6 and CRP has been reported to be associated with increased risk of CAD in healthy individuals. Here we show that serum levels of sgp130, binding partner of IL‐6, in highest quintile is associated with increased risk of CAD. In our ridge regression, we observed that are controlled by TNF receptors (CRP, sgp130, sICAM‐1, MMP2, and MMP1) contributes to the Lp, suggesting a bigger role of TNF‐a and IL‐6 as risk factors for CAD reported by earlier studies.^27^

## 5 CONCLUSIONS

T1D patients had higher serum levels of sTNFRI, sTNFRII, IGFBP2, IGFBP6, IGFBP1, IGFBP3, CRP, and SAA even after adjusting for confounding co‐variates. Our risk scores based on combining serum protein concentrations can be used for stratifying T1D patients into risk groups for CAD. These findings could be utilized for new therapeutic strategies to prevent or treat CAD in addition to being a clinically useful screening tool for activated inflammation in diabetes patients.

## 6 LIMITATION

The most important contribution of our study is the application of a machine‐learning approach to improve the accuracy of risk stratification for CAD in T1D patients through the Lp model. The 22 proteins evaluated looked a wide range of pathogenic pathways, including molecules involved in systemic inflammation, activation of inflammation, insulin‐like growth factor signaling, and vascular functioning. The breadth of serum markers, coupled with the large CAD population (*n* = 115), enhance the replicability of the results. The study is limited by its cross‐sectional nature. Classification of CAD was merely based on chart review and did not include further clinical evaluation. Although age was considered as a covariate during analysis, CAD patients were older than nCAD patients on average. Aging is associated with increased inflammation.^16^

## AUTHOR CONTRIBUTIONS

Carol Beatty, Katherine P. Richardson, and Sharad Purohit were involved with conception of the project. Sharad Purohit acquired the Luminex data. Sharad Purohit, Paul M. H. Tran, and Katherine P. Richardson were responsible for data analysis. Carol Beatty and Katherine P. Richardson wrote the initial draft of the manuscript. Ashok Sharma, Diane Hopkins, and Melissa Gardiner, contributed to clinical information and samples. All authors contributed to writing and editing of the manuscript.

## CONFLICT OF INTEREST STATEMENT

The authors declare no conflict of interest.

## Supporting information

Supporting information.

## Data Availability

All the research data presented in the study are included as figures or supplementary materials. Minimum data required to generate the figures and tables and the scripts used are available on Zenodo. org under the DOI https://doi.org/10.5281/zenodo.8169811.
