# Supplementary material for: Multiplex analysis of inflammatory proteins associated with risk of coronary artery disease in type‐1 diabetes patients
Source: Clin Cardiol. 2023 Oct 11;47(1):e24143. doi: 10.1002/clc.24143 (PMC10768730; doi:10.1002/clc.24143)
Supplement: Supplementary file 1 — Supporting information. [file CLC-47-e24143-s001.docx]

Supplementary Material

Article Title

Carol Beatty^1†^, Katherine P. Richardson^2,3†^, Paul M H Tran^2,4^, Khaled Bin Satter^2^, Diane Hopkins^2^, Melissa Gardiner^2^, Ashok Sharma^2^, Sharad Purohit^2,3,5,6*^

*** Correspondence:** Sharad Purohit: spurohit@augusta.edu

**Table S1:** Mean serum levels (Log2 transformed) of individual proteins between two groups. The p-values were adjusted according to the method described by Benjamini-Hochberg.

| **Protein** | **nCAD** | **CAD** | **FC** | **p** | **adpval** |
| --- | --- | --- | --- | --- | --- |
| IL1Ra | 8.34+1.64 | 8.46+1.56 | 1.09 | 4.85E-01 | 0.5081 |
| IL8 | 1.81+2.47 | 1.72+2.03 | 0.94 | 7.20E-01 | 0.72 |
| MCP1 | 5.98+0.65 | 6.08+0.5 | 1.07 | 6.39E-02 | 0.08786 |
| MIP1B | 5.1+1.86 | 4.81+1.66 | 0.82 | 1.16E-01 | 0.15012 |
| CRP | 23.04+2.48 | 24.2+2.54 | 2.24 | 2.07E-05 | 5.69E-05 |
| IGFBP1 | 10.14+1.7 | 10.84+1.49 | 1.62 | 4.39E-06 | 1.61E-05 |
| IGFBP2 | 12.46+2.4 | 14.45+1.9 | 3.96 | 1.21E-19 | 1.33E-18 |
| IGFBP3 | 23.75+0.88 | 23.3+1.47 | 0.73 | 3.04E-03 | 0.00608 |
| IGFBP6 | 16.03+0.93 | 16.84+0.77 | 1.75 | 8.38E-20 | 1.33E-18 |
| MMP1 | 9.28+1.49 | 9.89+1.24 | 1.53 | 1.82E-06 | 8.01E-06 |
| MMP2 | 15.48+0.69 | 15.74+0.57 | 1.2 | 1.04E-05 | 3.27E-05 |
| MMP9 | 17.67+1.16 | 17.34+1.54 | 0.8 | 3.72E-02 | 0.05846 |
| SAA | 23.69+2.48 | 24.46+2.44 | 1.7 | 2.84E-03 | 0.00608 |
| sEGFR | 16+0.72 | 15.88+0.65 | 0.92 | 5.86E-02 | 0.08595 |
| sgp130 | 19.36+0.8 | 19.49+1.06 | 1.1 | 2.15E-01 | 0.26278 |
| sICAM1 | 18.85+0.79 | 19.07+0.86 | 1.16 | 1.46E-02 | 0.02471 |
| sIL2Ra | 8.57+0.92 | 8.87+0.96 | 1.23 | 1.35E-03 | 0.0033 |
| sIL6R | 14.79+0.92 | 14.85+0.67 | 1.05 | 3.47E-01 | 0.3817 |
| sTNFRI | 8.77+0.79 | 9.6+0.97 | 1.77 | 6.48E-15 | 3.56E-14 |
| sTNFRII | 13.25+0.87 | 13.99+0.83 | 1.68 | 4.30E-16 | 3.15E-15 |
| sVCAM1 | 21.36+0.9 | 21.5+1.2 | 1.1 | 2.76E-01 | 0.31958 |
| tPAI1 | 16.28+0.89 | 16.01+0.9 | 0.83 | 3.66E-03 | 0.00671 |

*Adpval: Adjusted p-values, nCAD: T1D patients without coronary artery disease, CAD: T1D patients with coronary artery disease, FC: fold change derived by mean level of nCAD/mean level of CAD group

**Table S2:** Correlations between serum levels of proteins with age at which sample was drawn in non-CAD (nCAD, n=1107) and CAD (n=115) group.

|  | **nCAD (n=1107)** | | **CAD (n=115)** | |
| --- | --- | --- | --- | --- |
| **Protein** | **r** | **p-value** | **r** | **p-value** |
| IL1Ra | -0.0529 | 0.095 | -0.0008 | 0.994 |
| IL8 | -0.173 | 1.12E-07 | -0.0896 | 0.409 |
| MCP1 | -0.058 | 0.067 | 0.1271 | 0.220 |
| MIP1B | -0.1393 | 1.756E-05 | -0.0377 | 0.723 |
| CRP | 0.23307 | 3.10E-14 | 0.05534 | 0.583 |
| IGFBP1 | 0.02186 | 0.468 | 0.05815 | 0.537 |
| IGFBP2 | 0.24083 | 5.11E-16 | 0.20912 | 0.025 |
| IGFBP3 | -0.1426 | 4.13E-06 | 0.07349 | 0.463 |
| IGFBP6 | 0.27833 | 4.51E-21 | 0.14291 | 0.128 |
| MMP1 | 0.18908 | 2.45E-10 | 0.05519 | 0.558 |
| MMP2 | -0.1845 | 6.72E-10 | 0.2046 | 0.028 |
| MMP9 | -0.014 | 0.654 | 0.05055 | 0.616 |
| SAA | 0.11136 | 3.31E-04 | 0.08919 | 0.373 |
| sEGFR | -0.1071 | 3.64E-04 | -0.1366 | 0.149 |
| sgp130 | -0.0408 | 0.190 | 0.19808 | 0.047 |
| sICAM1 | -0.1751 | 1.56E-08 | 0.18203 | 0.070 |
| sIL2Ra | -0.2936 | 2.28E-23 | 0.04026 | 0.669 |
| sIL6R | -0.0494 | 0.1011222 | -0.0561 | 0.554 |
| sTNFRI | -0.1043 | 0.001 | 0.10938 | 0.245 |
| sTNFRII | 0.02719 | 0.366 | 0.20447 | 0.029 |
| sVCAM1 | -0.1386 | 7.65E-06 | 0.18697 | 0.063 |
| tPAI1 | -0.1581 | 3.78E-07 | -0.092 | 0.346 |

p-values were not adjusted for multiple testing, nCAD: T1D patients without coronary artery disease, CAD: T1D patients with coronary artery disease.

**Table S3:** Pearson Correlation (r) with the duration of type 1 diabetes.

|  | **nCAD (n=1107)** | | **CAD (n=115)** | |
| --- | --- | --- | --- | --- |
| **Protein** | **r** | **p-value** | **r** | **p-value** |
| IL1Ra | -0.037 | 0.243 | 0.138 | 0.198 |
| IL8 | -0.157 | 1.555E-06 | -0.091 | 0.399 |
| MCP1 | 0.003 | 0.913 | 0.066 | 0.527 |
| MIP1B | -0.079 | 0.015 | 0.173 | 0.101 |
| CRP | 0.180 | 5.11E-09 | -0.048 | 0.634 |
| IGFBP1 | 0.134 | 8.01E-06 | 0.177 | 0.058 |
| IGFBP2 | 0.274 | 2.09E-20 | 0.210 | 0.024 |
| IGFBP3 | -0.113 | 2.73E-04 | -0.168 | 0.092 |
| IGFBP6 | 0.236 | 1.99E-15 | 0.046 | 0.622 |
| MMP1 | 0.100 | 8.43E-04 | 0.134 | 0.155 |
| MMP2 | -0.086 | 0.004 | 0.290 | 0.002 |
| MMP9 | 0.0130 | 0.675 | -0.043 | 0.672 |
| SAA | 0.061 | 0.052 | -0.0068 | 0.946 |
| sEGFR | -0.028 | 0.357 | 0.056 | 0.554 |
| sgp130 | 0.002 | 0.941 | -0.045 | 0.656 |
| sICAM1 | -0.094 | 0.00265 | -0.156 | 0.122 |
| sIL2Ra | -0.159 | 1.18E-07 | 0.122 | 0.193 |
| sIL6R | -0.016 | 0.586 | -0.029 | 0.756 |
| sTNFRI | -0.035 | 0.251 | 0.038 | 0.687 |
| sTNFRII | 0.097 | 1.18E-03 | 0.059 | 0.536 |
| sVCAM1 | -0.081 | 0.0089 | -0.128 | 0.204 |
| tPAI1 | -0.092 | 3.17E-03 | -0.012 | 0.899 |

p-values were not adjusted for multiple testing, nCAD: T1D patients without coronary artery disease, CAD: T1D patients with coronary artery disease

**Table S4:** Differences in serum levels of proteins between two genders.

|  | **nCAD (n=1107)** | | **CAD (n=115)** | |
| --- | --- | --- | --- | --- |
| **Protein** | **F/M** | **p** | **F/M** | **p** |
| IL1Ra | 1.19 | 0.017058 | 1.50 | 0.083 |
| IL8 | 0.75 | 0.012 | 0.75 | 0.354 |
| MCP1 | 0.92 | 0.00398 | 0.98 | 0.737 |
| MIP1B | 0.76 | 0.00124 | 1.52 | 0.088 |
| CRP | 1.80 | 5.61E-08 | 2.89 | 0.00254 |
| IGFBP1 | 1.00 | 0.962 | 1.28 | 0.203 |
| IGFBP2 | 0.99 | 0.943 | 0.78 | 0.308 |
| IGFBP3 | 1.10 | 0.01644 | 1.15 | 0.499 |
| IGFBP6 | 0.90 | 4.34E-03 | 0.82 | 0.042 |
| MMP1 | 1.07 | 0.263 | 0.97 | 0.863 |
| MMP2 | 0.94 | 0.027 | 0.93 | 0.369 |
| MMP9 | 1.09 | 0.080 | 0.82 | 0.366 |
| SAA | 2.20 | 2.13E-13 | 1.89 | 0.057 |
| sEGFR | 0.96 | 0.147 | 0.93 | 0.429 |
| sgp130 | 0.94 | 0.057 | 0.90 | 0.458 |
| sICAM1 | 0.93 | 0.043 | 0.96 | 0.756 |
| sIL2Ra | 0.91 | 0.015 | 0.89 | 0.372 |
| sIL6R | 1.01 | 0.813 | 1.11 | 0.225 |
| sTNFRI | 0.93 | 0.01220 | 0.91 | 0.457 |
| sTNFRII | 1.04 | 0.299 | 1.13 | 0.270 |
| sVCAM1 | 0.91 | 0.020 | 0.95 | 0.740 |
| tPAI1 | 0.96 | 0.339 | 1.10 | 0.434 |

p-values were not adjusted for multiple testing, nCAD: T1D patients without coronary artery disease, CAD: T1D patients with coronary artery disease, F: females, M:males


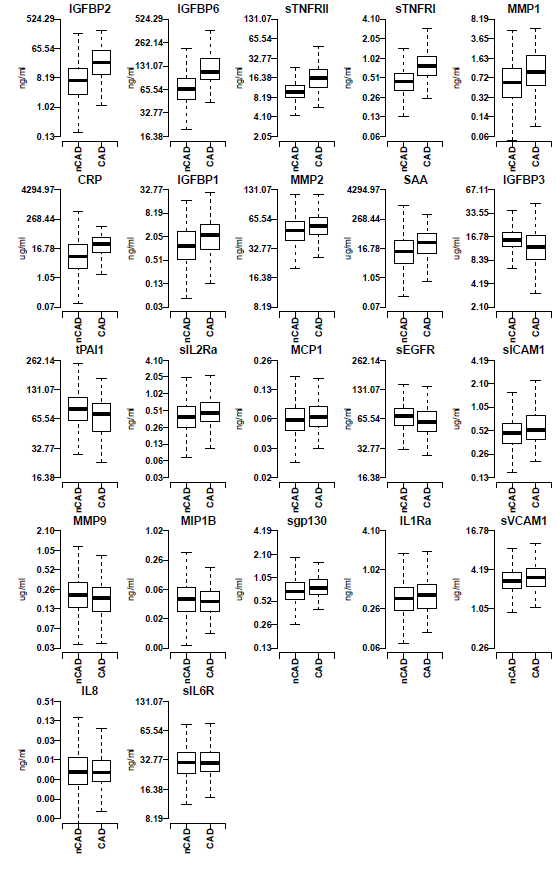


**Figure S1:** Boxplots showing distribution of serum levels of proteins in T1D patients with coronary artery disease(CAD) and without (nCAD). Concentration values are plotted on the y-axis, and groups are on the x-axis. Log2 transformed concentration values are plotted initially, then the y-axis is populated with back transformed concentration. Univariate differences were tested using t-tests (refer Table S1 for pvalues). ug:microgram, ng:nanogram


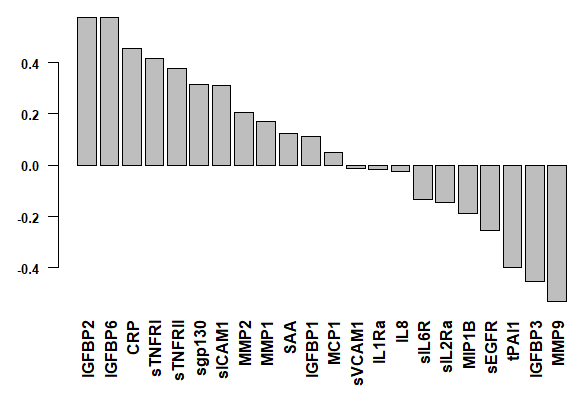


**B**

**A**


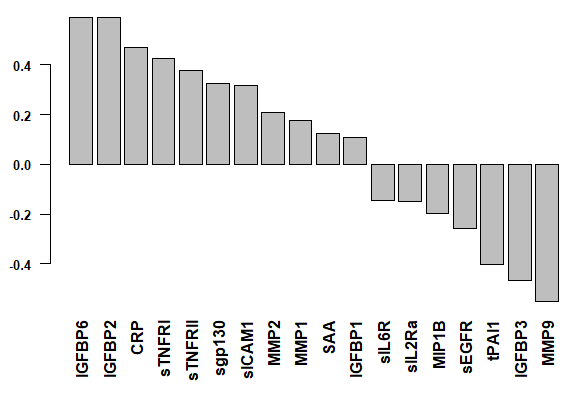


**Figure S2: Barplots of ridge regression coefficients (relative contribution) of individual proteins used to generate linear predictor (Lp) score.** The Lp score is a sum of the values of individual proteins multiplied by their respective relative contribution. Proteins that contributes least were dropped in each step to generate Lp using 22 proteins (A) and 18 proteins (B). Prior to ridge regression, log 2 concentration data was scaled to unit SD and then subjected to ridge regression.


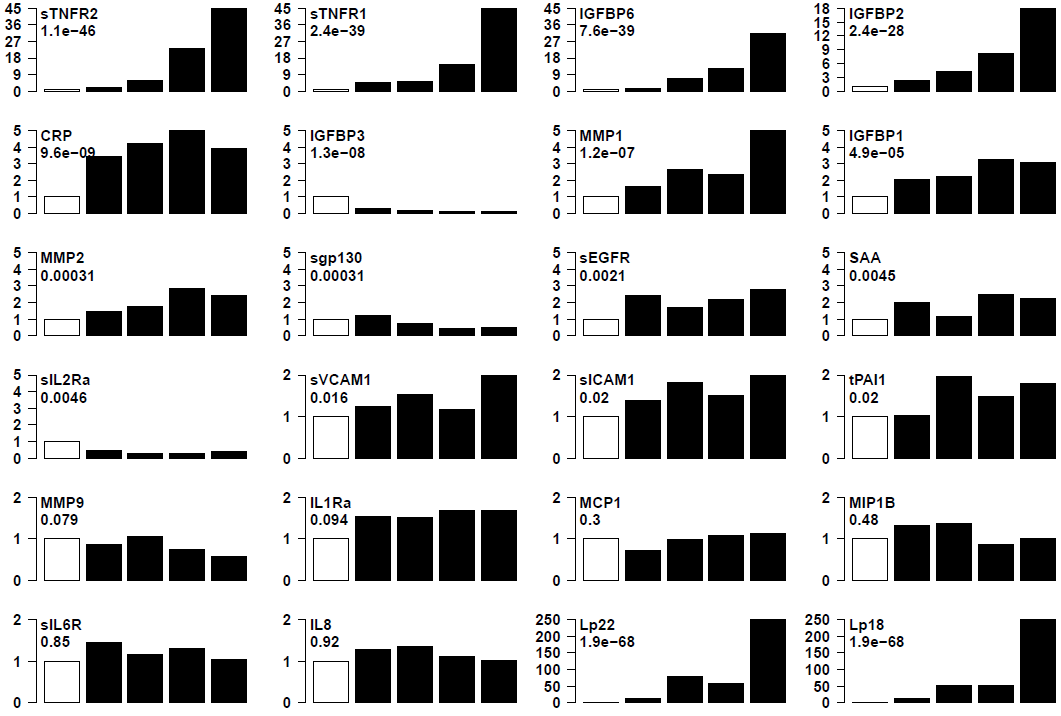


**Figure S3:** Odds ratios (Y-Axis) associated with each of the top four quintiles (20 percentile) compared to the bottom 1^st^ quntile for each of the 22 individual proteins. Open bar represents the 1^st^ quintile used as a reference (OR=1). Each of the solid bars represents 2^nd^ to 5^th^ quintiles (20% of CAD patients)
